# A Low-Cost Handheld Centrifugal Microfluidic System for Multiplexed Visual Detection Based on Isothermal Amplification

**DOI:** 10.3390/s24155028

**Published:** 2024-08-03

**Authors:** Nan Wang, Xiaobin Dong, Yijie Zhou, Rui Zhu, Luyao Liu, Lulu Zhang, Xianbo Qiu

**Affiliations:** Institute of Microfluidic Chip Development in Biomedical Engineering, College of Information Science and Technology, Beijing University of Chemical Technology, Beijing 100029, China

**Keywords:** centrifugal microfluidic, RPA, handheld, multiplexed, visual detection

## Abstract

A low-cost, handheld centrifugal microfluidic system for multiplexed visual detection based on recombinase polymerase amplification (RPA) was developed. A concise centrifugal microfluidic chip featuring four reaction units was developed to run multiplexed RPA amplification in parallel. Additionally, a significantly shrunk-size and cost-effective handheld companion device was developed, incorporating heating, optical, rotation, and sensing modules, to perform multiplexed amplification and visual detection. After one-time sample loading, the metered sample was equally distributed into four separate reactors with high-speed centrifugation. Non-contact heating was adopted for isothermal amplification. A tiny DC motor on top of the chip was used to drive steel beads inside reactors for active mixing. Another small DC motor, which was controlled by an elaborate locking strategy based on magnetic sensing, was adopted for centrifugation and positioning. Visual fluorescence detection was optimized from different sides, including material, surface properties, excitation light, and optical filters. With fluorescence intensity-based visual detection, the detection results could be directly observed through the eyes or with a smartphone. As a proof of concept, the handheld device could detect multiple targets, e.g., different genes of African swine fever virus (ASFV) with the comparable LOD (limit of detection) of 75 copies/test compared to the tube-based RPA.

## 1. Introduction

Polymerase chain reaction (PCR) technology is well known for its high sensitivity and accuracy [1]. However, the application of PCR to rapid diagnosis is limited due to the high system complexity, high cost and long detection time [2,3]. In comparison, isothermal amplification technology (IAT) has the advantages of low, constant heating temperature, short reaction time and easy operation [4,5]. The integration of isothermal amplification methods with microfluidic systems has the potential to establish a reasonable point of care (POC) analysis platform [6,7,8].

Different from single-target detection, multiple targets can be detected in parallel on multiplexed microfluidic chips to save time and cost with high efficiency [9,10]. Ultra-fast DNA-based multiplex detection with convection PCR was developed for meat species identification in on-site applications [11]. Jin et al. reported a LAMP-based microfluidic platform that enables simultaneous detection of multiple waterborne pathogens with one-time sample loading [12]. Rapid and sensitive multiplex molecular diagnosis of respiratory pathogens was achieved with a plasmonic isothermal RPA array chip [13].

Different centrifugal microfluidic systems have been developed for multiplexed detection [14,15,16]. Based on centrifugal actuation, efficient mixing can be conveniently achieved by a metal bead or Euler force inside the reaction chamber, which is helpful in improving RPA detection sensitivity [5]. A real-time centrifugal microfluidic system based on RPA was developed for multiple detection of pathogenic microorganisms of penaeid shrimp [17]. Due to the bulky and complicated servo motor system which is used for precise and high-speed rotation, for most existing centrifugal microfluidic platforms, it is difficult to shrink their size to a smaller one, e.g., handheld size [18]. On the other side, due to real-time fluorescence detection, the cost of centrifugal microfluidic platforms is relatively high [14]. As one of the choices for POC diagnosis, low-cost visual IAT is attractive to simple on-site testing [4,19,20]. Wei et al. developed a portable, rapid, accurate and easy-to-operate diagnosis device based on RPA with visual detection [21]. By taking advantage of visual detection, in principle, the cost of a microfluidic system can be reduced with the simplified optical module. 

To achieve efficient, convenient and parallel diagnosis, portable, simple, low-cost, easy-to-operate and multiplexed devices are highly desired in resource-limited environments, such as the countryside with poor medical facilities [22]. Different from most existing, bulky and expensive centrifugal multiplexed microfluidic systems, here, we developed a low-cost, compact, and handheld microfluidic centrifugation platform for multiplexed visual detection based on isothermal amplification. A centrifugal microfluidic chip consisting of four reaction units was developed for multiplexed detection. In the design of low-cost and hand-held microfluidic devices, capillary action and finger actuation are commonly used for sample injection and mixing. However, capillary action often requires hydrophilic treatment of the microfluidic channels. In the multi-channel microfluidic chips, it is difficult to achieve parallel distribution of the samples simply with capillary force. To achieve more uniform sample distribution in each reaction unit for multi-target detection, an elaborate fluid actuation mechanism is essential. While finger-driven methods can be utilized for sample injection and mixing, their accuracy of fluid control is limited, and it is difficult to perform continuing, repetitive actuation by fingers. The centrifugal microfluidic system driven by a small DC motor is capable of simultaneously performing one-time sample injection and repeatedly mixing through motor rotation speed control. Different from finger-based actuation, highly consistent, elaborate and reliable fluid control can be achieved by centrifugation-based actuation. Therefore, a small DC motor controlled by a flexible custom strategy, and meanwhile, simplified visual detection with optimized performance, were adopted to significantly shrink both the size and cost of the handheld device. Automatic sample distribution, efficient heating, active mixing, parallel amplification, and convenient visual detection have been fully integrated into the handheld device. The functionality and performance of the handheld microfluidic platform have been demonstrated by successfully detecting different ASFV genes with reasonable sensitivity.

## 2. Materials and Methods

### 2.1. Centrifugal Microfluidic Chip

**Figure 1:**
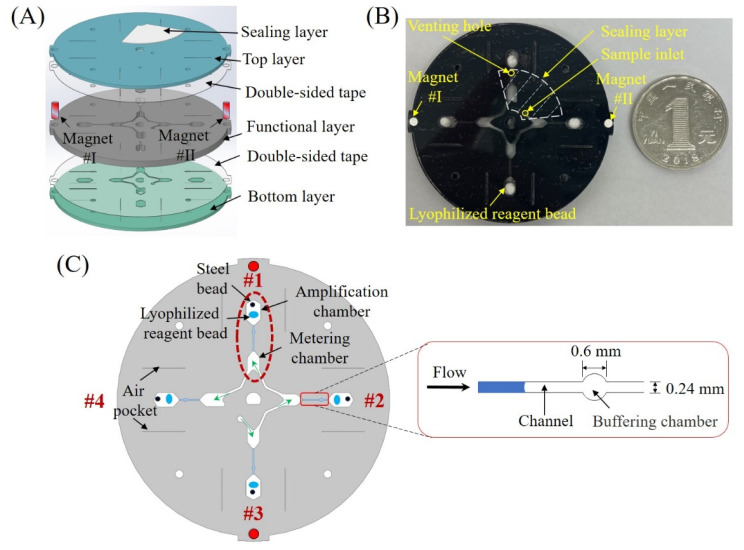
Centrifugal microfluidic chip for multiplexed nucleic acid diagnosis: (**A**,**B**) an exploded view and a picture of the chip, respectively; (**C**) enlarged diagram of the chip layout with four reaction units.

As shown in Figure 1A, the centrifugal microfluidic chip consists of three different layers (top, middle functional, and bottom layers). Both the top and the bottom layers are made from 1 mm transparent polymethyl methacrylate (PMMA), and the middle layer is made from 2 mm black PMMA. Each two adjacent layers are bonded together with 150 μm thick double-sided adhesive tape (300LSE, 3M). Different double-sided adhesive tapes have been used in the fabrication of various microfluidic chips. And their biocompatibility had already been confirmed by other related research [23]. Moreover, in our previous research of the centrifugal microfluidic system, the same double-sided adhesive tapes were used to fabricate a microfluidic chip for RPA detection, and it showed that the biocompatibility of the used double-sided adhesive tape is desirable for RPA amplification [5]. Two small magnetic parts (magnet #I and #II) are mounted on the border of the chip, which is used for positioning control. As shown in Figure 1B, the black chip is similar to other typical centrifugal chips but with a quite small size (diameter: 52 mm, thickness: 4 mm). Four white lyophilized RPA reagent beads are pre-stored inside four amplification chambers, respectively. Once the test sample is loaded into the chip, the sample inlet and the venting hole can be sealed by a sealing layer from the top (as shown in Figure 1A,B). After sample loading, single-sided adhesive tape was used to seal all the inlets and outlets of the microfluidic chip to prevent contamination and ensure consistent conditions for amplification.

As shown in Figure 1C, there totally have four independent reaction units (#1–#4) and each one consists of a metering and downstream amplification chamber. As indicated by four center arrows in Figure 1C, totally four connected metering chambers form a one-way sample introducing path (from the sample inlet to the venting hole). With one-time sample loading, four metering chambers can be consecutively filled. To prevent the sample from entering the amplification chamber during sample introduction, a narrow channel (depth: 0.4 mm, width: 0.24 mm) with a small buffering chamber (depth: 0.4 mm, diameter: 0.6 mm) is inserted between the metering and amplification chambers to increase flow resistance, which conversely can also avoid reagent leakage from the amplification chamber in reaction. The size of the first three metering chambers is ~25 μL, and the last one has a larger size, e.g., ~30 μL to hold excessive samples. After the RPA mix was loaded into the metering chamber, the DC motor was needed to rotate to transfer the liquid in each metering chamber into the corresponding amplification chamber with high-speed rotation; for example, in our case, approximately 3000 rpm. The rotation speed itself did not affect the amplification effect. The rotation speed of the DC motor affects liquid transferring, which may affect amplification for example when the chamber is not fully filled by reagent. The inner surface of each metering chamber and the channel between each two of them are coated with hydrophilic reagent (2% Tween-20 in ethanol) to enable smooth fluid flow. In each amplification chamber, a tiny steel bead (diameter: 1 mm) is pre-stored with the lyophilized RPA reagent for active mixing. The size of each amplification chamber is designed as ~26 μL witnessing the small embedded mixing bead. To reduce energy loss during heating, double sides of each amplification chamber are surrounded by two air pockets, respectively. Once the test sample enters the amplification chamber, the pre-stored dry reagent will be hydrated for amplification. An isothermal amplification kit (Weifang Amp Future Biotech Co., Ltd., Weifang, China) was adopted to perform RPA amplification, which mainly included buffers A and B, and the lyophilized reagents. However, the target gene sequences and the specific primer sequences were confidential and cannot be provided. The performance evaluation of lyophilized reagents was performed by the reagent supplier, and it was found that the performance between the lyophilized reagents and non-lyophilized reagents was comparable.

The following is the procedure of reagent and sample preparation, and chip test for single target detection. (1) 5 μL of plasmid DNA template containing the P72 gene, 14.7 μL of buffer A, 1.25 μL of buffer B and 4.05 μL of sterile water were added into a test tube, which achieved a total size of 25 µL. (2) After completely mixing, the RPA mixture was loaded into a single reaction chamber and pre-stored with dry RPA reagent. The no-primer control was used for the negative control test.

### 2.2. Handheld Companion Device

**Figure 2:**
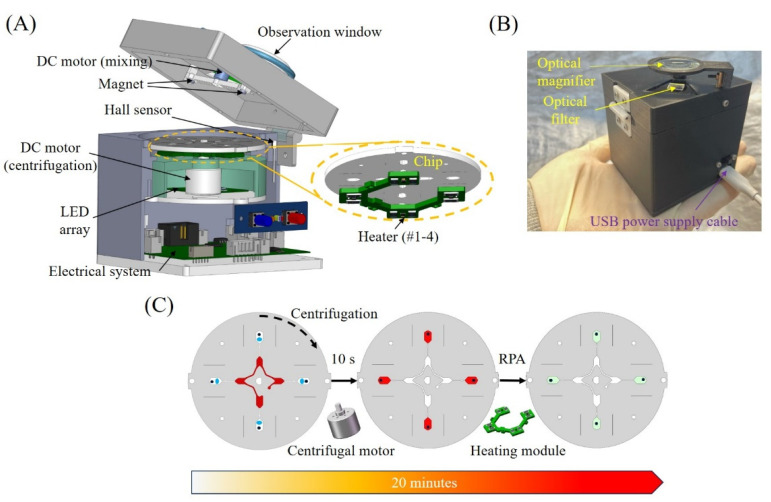
Handheld companion device for multiplexed detection: (**A**) schematic design of the handheld device consisting of different modules; (**B**) a picture of the device; (**C**) chip actuation with the handheld device for multiplexed detection within 20 min.

As shown in Figure 2A, the fully integrated handheld device consists of non-contact heating, optical, rotation and sensing modules, which are able to perform different tasks with the microfluidic chip for multiplexed detection. The main challenges of device miniaturization can be included as follows: (1) miniaturization design of the optical module for visual fluorescence detection; (2) miniaturization design of the heating module; (3) highly-compact integration of mechanical and optical components; (4) miniaturization of the centrifugal microfluidic chip. 

A custom heating module, which consists of four independent heaters (#1–4), respectively, for four amplification chambers, is located below the chip with an air gap of 0.8 mm. The rotation module consists of two small DC motors, and one of them (614 coreless DC motor with 3.3 V power supply) is mounted on top of the chip to introduce active mixing into the highly viscous reagent by iteratively actuating the steel bead with magnetic force, while the other one (15 mm coreless DC motor with 3.3 V power supply) is mounted below the chip to perform centrifugation and positioning. For typical centrifugal microfluidic systems, normally an expensive and advanced servo motor system with a large size is adopted to achieve both high-speed centrifugation and accurate positioning with feedback control. Here, to reduce both the size and cost of the handheld device, a small DC motor was adopted for centrifugation. Accordingly, a magnetic sensing module was developed to guide accurate chip positioning with magnetic locking, e.g., switch between different working positions. As shown in Figure 2B, the size of the handheld device is ~72 mm × 65 mm × 68 mm, and most of its mechanical components are fabricated with 3D printing. The electrical system of the handheld device is controlled by a 32-bit microcontroller unit (GD32F350), which performs a couple of different functions, including PID temperature control, LED powering, DC motor driving, magnetic sensing and operator interfacing. As shown in Figure 2A, each amplification chamber is heated by two 1.5 W resistors (33 ohms, 2010) as a heater unit (#1–4), and TMP112AIDRLR is used for temperature measurement. Except for optical filters, the total cost of system components is $15.

As shown in Figure 2C, after the sealed chip with pre-loaded sample is put into the handheld device, the test sample in each metering chamber can be driven into the downstream chamber by high-speed centrifugation within 10 s. After that, the chip is re-located and locked to allow each amplification chamber to sit on top of one heater, and then, non-contact heating is initiated for 20-min amplification before visual observation. To effectively achieve rapid on-site detection, there were two modes for power supply for the handheld device. First, the handheld device can be powered by an outside adapter with a type-C interface (Voltage: 5 V, Current: 2 A). Second, an 18,650-lithium battery (Voltage: 3.7 V, Capacity: 3000 mAh), which supports two runs, can also be used as another power supply. Two power supplies can be automatically switched just like other electrical devices.

### 2.3. Chip Positioning Based on Magnetic Sensing and Locking

**Figure 3:**
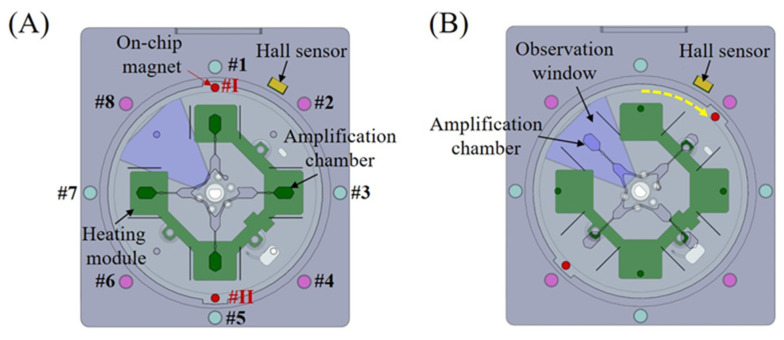
Chip positioning based on magnetic sensing and locking: (**A**) heating position for parallel RPA amplification; (**B**) detection position for visual observation.

Instead of using the complicated feedback control of traditional servo motor systems, accurate chip positioning, e.g., switching between heating and detection positions, is achieved with the assistance of the custom magnetic sensing mechanism. As shown in Figure 3A, the magnetic sensing module consists of a Hall sensor and eight equally distributed outside magnetic parts (#1–8) with a 45° angle distance, which defines eight different positions (heating or detection). Another two on-chip magnetic parts (#I, #II) in the same diameter direction work with the Hall sensor and the outside magnetic parts to achieve accurate chip positioning. When the rotation speed of the chip becomes smaller after the centrifugal DC motor is powered off, one outside and on-chip magnetic parts will work together to lock the chip at one of eight different positions due to the magnetic force between them. As shown in Figure 3B, the centrifugal DC motor can be powered on and off iteratively within a short time for small rotation until one on-chip magnetic part triggers the Hall sensor at the end of rotation before it is caught by #2 outside magnetic part, which will lock the chip on the detection position. After that, to switch from the detection to the heating position (as shown in Figure 3A), the centrifugal DC motor is powered on at a low speed for a short time until one on-chip magnetic part is caught by #3 outside magnetic part. To allow each amplification chamber to be visually observed by eyes in turn, a similar actuation mechanism can be adopted to switch the chip status between the detection and the heating positions iteratively.

### 2.4. Visual Detection

**Figure 4:**
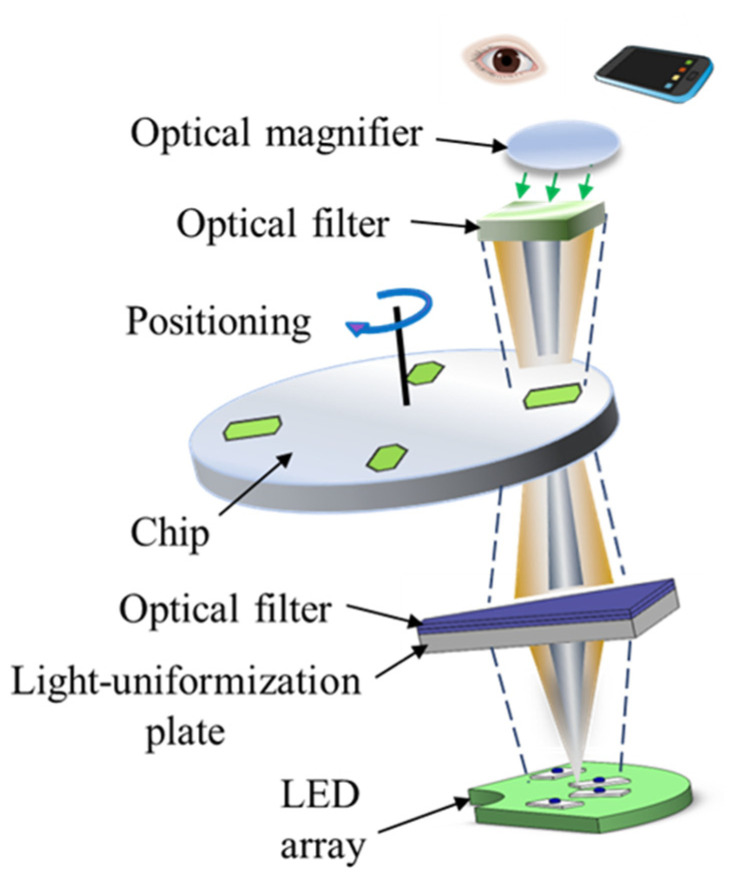
Schematic illustration of visual detection.

As shown in Figure 4, after amplification, the detection result of each amplification chamber can be readily observed by eyes through the optical module. The amplicons inside the reaction chamber are excited by an LED array through a band-pass optical filter with a light-uniformization plate (75% transmittance, Xiyangyang Acrylic Plexiglass Products Factory, Shenzhen, China) from the bottom. Instead of using a single LED, an LED array with four blue LEDs (XL-2835UBC-05, Shenzhen Chengxingguang Electronics Technology Co., Ltd., Shenzhen, China) is adopted to illuminate the reagent to achieve more uniform fluorescence signal distribution with higher intensity. The light-uniformization plate is adopted to uniformize excitation light from four LEDs. Another band-pass optical filter is located on top of the ‘observation window’ for fluorescence signal observation by eyes from outside. To enable clearer observation, a small optical magnifier can be mounted on top of the optical filter. With magnetic sensing, the chip is re-located and locked to allow each amplification chamber to reach the ‘observation window’ one by one. In addition, the choice of materials and the coating methods of the double-sided adhesive were decided based on the performance of visual observation. The surface condition of different materials may affect the performance of visual observation. The material with a smooth surface could enhance the reflection of the excitation light, thereby deteriorating visual observation. Conversely, the matte surface could effectively reduce reflected light which could affect visual observation. Also, surface-coating to the double-sided adhesive tape is helpful to minimize the scattering of excitation light, as well as to reduce the background fluorescence from the double-sided tape. As shown in Figure 4, alternatively, each amplification chamber can also be detected by an outside smartphone, which will provide quantitative discrimination between positive and negative tests based on the signal intensity of the fluorescence image collected by the smartphone. A custom image processing software running on a smartphone was developed to extract the gray value of the fluorescence image for each amplification chamber, and the positive test can be discriminated from the negative test by comparing their gray values. As a compatible method with eye-based visual detection, smartphone-based detection can be conveniently implemented to perform more objective analysis especially when the signal intensity difference between positive and negative tests is insignificant in POC testing.

## 3. Results and Discussion

### 3.1. Optimization of Visual Detection with Improved Performance and Limited Cost

To achieve the desired performance of visual detection through significant and straightforward discrimination between positive and negative tests based on the difference between their fluorescence intensity, much effort has been made in different respects, including analysis of background fluorescence which is relative to material, surface property, and excitation light, and as well as, configuration of different optical filters with different wavelengths and grade levels. 

**Figure 5:**
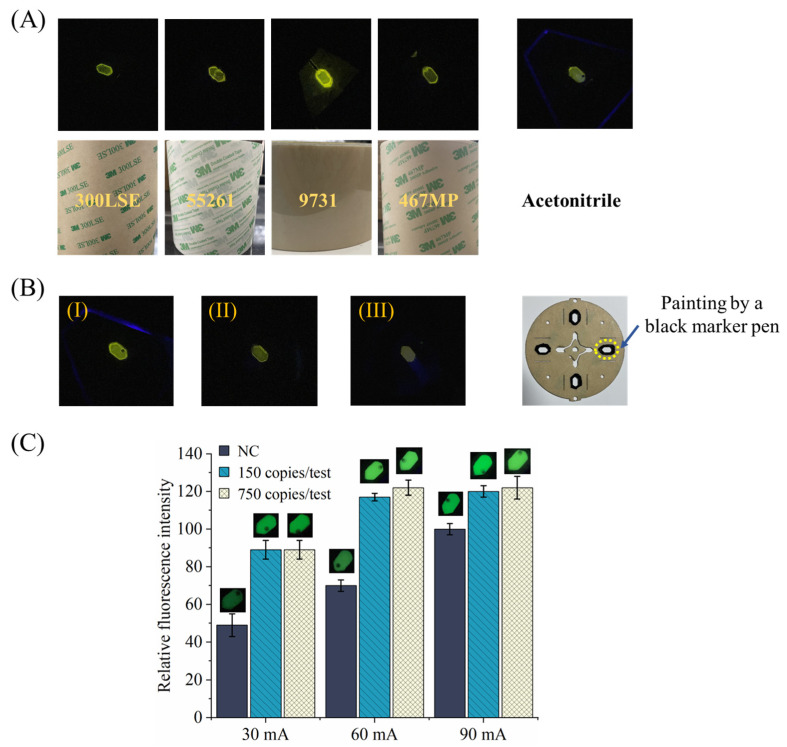
Strategy to reduce background fluorescence: (**A**) background fluorescence from different types of 3M double-sided tape, as well as from acetonitrile-bonding chip; (**B**) two ways to depress original background fluorescence (**I**), one is to paint double-sided tape with black material to block the background fluorescence (**II**), and the other is to use black bottom-frosted PMMA to reduce scattered light (**III**); (**C**) detection results with different LED working currents. (Inserted images with corresponding gray values are collected by smartphone).

As shown in Figure 5A, it has been found that, for different types of double-sided tape (300LSE-55261-9731-467MP, 3M) that are chosen for chip bonding, they emit background fluorescence with different signal intensity within a wavelength range that is quite close to the center wavelength of positive RPA amplicons. In contrast, for the chip bonded with acetonitrile, the signal intensity of background fluorescence around the boundary profile of the chamber is significantly lower than that of the chip bonded with double-sided tape. Meanwhile, for all the reaction chambers, background fluorescence can also be observed from the chamber center where there is no double-sided tape. Therefore, to depress the background fluorescence from the transparent PMMA itself, black PMMA is chosen for the middle functional layer of the centrifugal chip. 

As shown in Figure 5B, the background fluorescence from the PMMA reactor with double-sided-tape bonding (case (I)) can be weakened in at least two ways (case (II) and (III)). For case (II) in Figure 5B, the surface of the double-side tape around the boundary profile of the reaction chamber can be painted with a black marker pen (as shown in the last picture of Figure 5B), which significantly reduces the background fluorescence from the double-sided tape. For case (III) in Figure 5B, instead of using black PMMA with a bright surface, the black bottom-frosted PMMA was adopted to significantly reduce the background fluorescence caused by scattered light. 

Taqman probe labeled with FAM was used for fluorescence detection. In the RPA reagent, buffer A consisting of big molecules was used to stabilize the proteins in the reagent and maximize their activity. Buffer B consisting of Mg^2+^ ions was used to activate the enzyme for reaction. 

The working current of LEDs was regulated to find the proper status with a higher signal ratio of positive to negative tests for eye observation. As shown in Figure 5C, when the working current of single LED is 60 mA, the eye-observable difference between positive tests (150, 750 copies/test with P72 gene, the “copies/test” represents in each amplification run, the number of templates in the RPA mix. The reference volume of a test was 25 μL) and negative control (NC) are more significant than that with other two settings (30 mA, 90 mA), which is confirmed by the fluorescence signal intensity achieved with the smartphone-based detection. When the LED working current is too low, the fluorescence reagent cannot be thoroughly excited with desired efficiency, while when it is too high, more background fluorescence will be emitted from different materials; meanwhile, excessive scattered excitation light will escape from the optical filter, and both of them will deteriorate the detection performance. The experiment was repeated three times, and similar results were achieved. 

**Figure 6:**
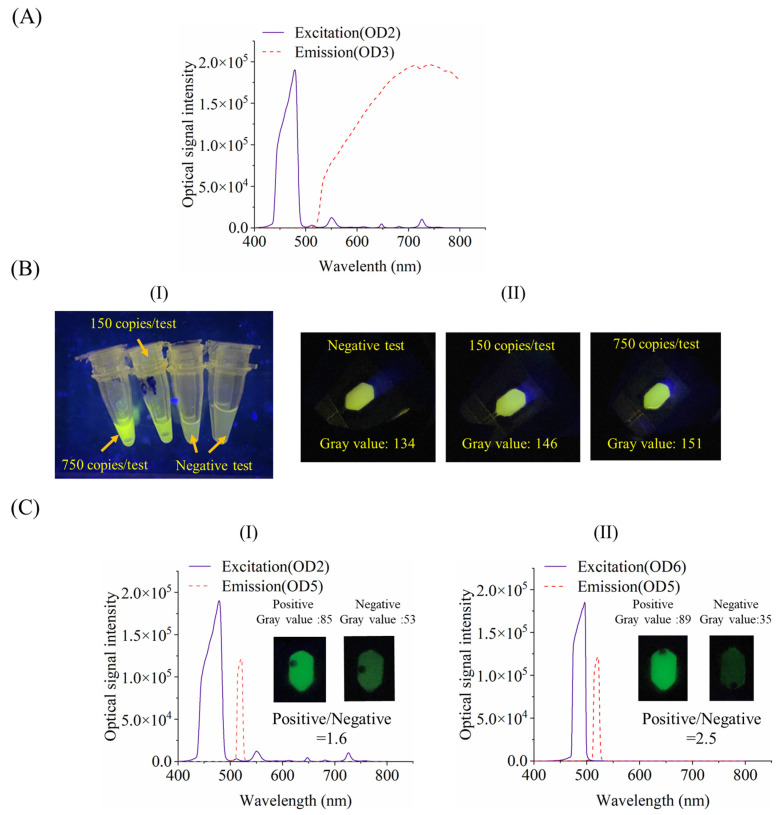
Visual detection under different conditions: (**A**) transmittance spectrums for both the excitation (OD2) and the emission (OD3) optical filters; (**B**) comparison between tube- (**I**) and chip-based visual detection (**II**); (**C**) comparison between different optical filter settings: (**I**) two OD2, OD5 optical filters for excitation and emission, respectively; (**II**) two OD6, OD5 optical filters for excitation and emission, respectively.

To find out the proper optical filters with economical cost, different settings of optical filters with different transmittance spectrums and grade levels (e.g., optical density, OD) were evaluated and compared. As shown in Figure 6A, to limit the device cost, one band-pass excitation (OD2, Gengxu photoelectricity Co. Ltd., Shenzhen, China) and long-pass emission optical filters (OD3, Gengxu photoelectricity Co., Ltd., Shenzhen, China) with relatively low values of optical density are first evaluated (all the transmittance spectrums were measure by QEPRO, Ocean Optics). When these cheap optical filters with low OD values and wide band ranges were adopted, two duplicated optical filters were used for excitation or emission. As shown in Figure 6(B-I), for tube-based tests, even with these cheap optical filters, positive tests (ASFV P72 gene, 150 and 750 copies/test) can be easily discriminated from the negative test with visual observation. In contrast, as shown in Figure 6(B-II), for chip-based test, with the same setting of small-size optical filters (OD2 for excitation (6 mm × 6 mm, $3), OD3 for emission (9 mm × 9 mm, $4)), positive tests (gray values for 150 and 750 copies/test are 146 and 151, respectively) cannot be creditably discriminated from the negative test (gray value is 134) because their gray values are quite close to each other. 

As shown in Figure 6C, to achieve optimal visual detection, different optical filters are adopted for evaluation. Besides negative control (NC), the PC gene (750 copies/test) was detected in experiments. As shown in Figure 6(C-I), instead of using the cheap long-pass emission filter (OD3), a more expensive band-pass filter (OD5, 9 mm × 9 mm, $10) is adopted to block the background fluorescence, which enables easy discrimination between positive and negative tests with a significant signal ratio of positive to negative tests (positive/negative = 1.6). As shown in Figure 6(C-II), when both the cheap excitation and emission filters are replaced by more expensive band-pass filters with sharp band ranges (OD6 for excitation (6 mm × 6 mm, $20), OD5 for emission (9 mm × 9 mm, $10)), the performance of visual detection can be further improved with an even higher signal ratio of positive to negative tests (positive/negative = 2.5).

It can be concluded that, for tube-based tests with low background fluorescence, desired visual detection can be conveniently achieved with even cheap optical filters. In contrast, for chip-based tests, due to the negative effect and the disturbance caused by high background fluorescence, more expensive optical filters need to be adopted to achieve satisfactory performance.

### 3.2. Multiplexed ASFV Detection with the Handheld Centrifugal Microfluidic System

The following is the protocol implemented on the microfluidic chip for multiplexed detection. A total of 21 µL of plasmid DNA containing two ASFV genes (P72 gene, P54 gene), PC gene (positive control), 61.75 µL of buffer A, and 5.25 µL of buffer B, 17 µL of water are pre-mixed, which achieve a total size of 105 µL before it is loaded into the chip. The ratio of the reagent combination used in this work was all the same. The difference was that the total volumes of PRA mix in different experiments were not the same. Considering the potential dead volume inside the microfluid chip which could be caused by different respects, for example, sample loading, and reagent distribution, the total RPA mix loaded into the microfluidic chip is 105 μL, instead of 100 μL which is equal to the total volume of four amplification chambers.

The fluidic mixing efficiency of the centrifugal microfluidic system has already been thoroughly studied and discussed in our previous research [5]. The results demonstrated that the centrifugal microfluidic chip with micro-bead-based mixing can achieve completely uniform mixing with a sample size of 50 μL within two seconds. After high-speed centrifugation, in each amplification chamber, the 25 µL reaction mix is ready for amplification. Multiplexed detection can be achieved by pre-storing different primers in different chambers.

**Figure 7:**
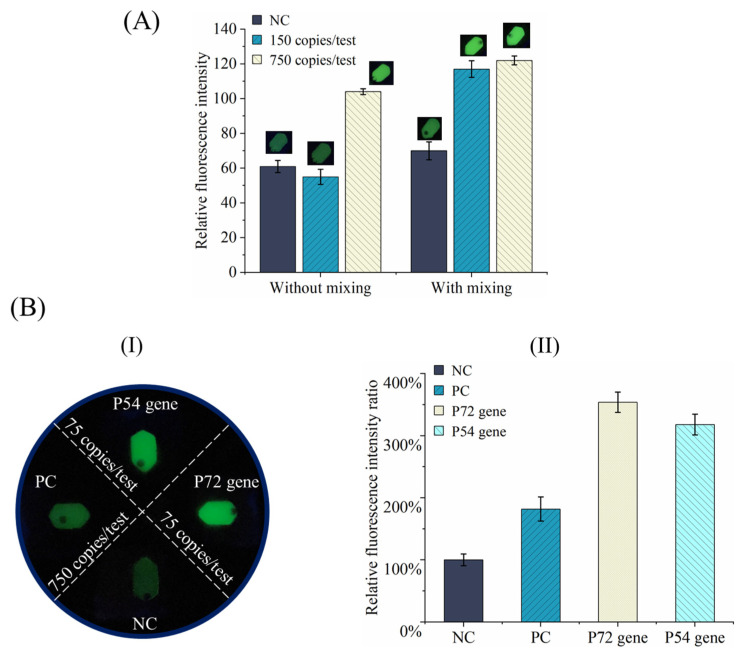
Performance evaluation of the handheld system for multiplexed detection: (**A**) comparison of the detection results between with and without active mixing (Inserted images with corresponding gray values are collected by smartphone.); (**B-I**) fluorescence pictures of four reaction chambers for multiplexed detection; (**B-II**) comparison of fluorescence signal intensity between positive and negative tests.

It has been proven that, without active mixing, the detection sensitivity could deteriorate because of the inhibition of the natural disassembly of recombinase primer due to the high viscosity of the RPA reagent [24]. Similar to our previously reported work [5], for the centrifugal chip, active mixing was introduced in amplification. A small DC motor was used to continually drive the tiny steel bead inside the chamber with two small permanent magnetic parts for 10 s after each 2 min in amplification. Besides negative control (NC), two different samples with different concentrations (750 and 150 copies/test) of the ASFV P72 gene were detected in experiments. As shown in Figure 7A of this work, with active mixing, both positive samples can be successfully discriminated from the negative test by observing and comparing fluorescence signals just with the eyes. In contrast, without active mixing, it can be found that the positive test with the low concentration of 150 copies/test cannot be discriminated from the negative test based on both visual detection and smartphone-based detection. The experiment was repeated three times, and similar results were achieved. 

All the reagents were provided by the supplier and their performance of specificity and sensitivity for multiplexed detection to different targets was systematically evaluated and confirmed by the manufacturer. For the microfluidic chip, its major goal is to automate and miniaturize the process of diagnosis and meanwhile to achieve comparable performance to the benchtop test. The quantitative data to support claims of sensitivity and specificity in ASFV gene detection has already been added in Figure 7. Multiple tests of samples with different concentrations, for example, from high to low, were performed in this study. The sensitivity was evaluated by the limit of detection (LOD). As shown in Figure 7(B-I), both P72 and P54 genes can be successfully discriminated from negative tests with the LOD of 75 copies/test based on visual observation. Notably, the fluorescence brightness of the PC sample is affected by both the amplification efficiency and the template concentration. For the PC control sample, the amplification efficiency is not fully optimized since it works as an internal control. Therefore, it is not recommended to perform quantitative analysis and comparison of the fluorescence brightness between PC control and target genes. As shown in Figure 7(B-II), the fluorescence signal intensity (achieved with smartphone-based detection) of all positive samples is significantly higher than that of the negative test. Each experiment was repeated three times, and similar results were achieved. 

The specificity in ASFV gene detection was evaluated by the multi-target amplification of the ASFV gene on the developed centrifugal microfluidic chip. Specifically, the test sample was equally distributed into four amplification chambers, and it was independently amplified with three different sets of primers, respectively, for PC, P72, and P54 genes in three reactors except one without primers for the negative control test. As shown in Figure 7(B-I), the amplification specificity was satisfied for each target, demonstrating reasonable selectivity in ASFV gene detection. For the P72 gene, the sensitivity achieved by the handheld device with single-stage amplification is lower than that of our previously developed real-time, multiplexed centrifugal microfluidic system with two-stage amplification (~7.5 copies/test) [5]. For the P54 gene, comparable detection sensitivity is achieved by our both devices. In contrast, for tube-based RPA, the reported LODs for the ASFV P72 gene are 17.5 copies/test [25] and 93.4 copies/test [26], respectively.

## 4. Conclusions and Outlook

To extend the application field of centrifugal microfluidic systems, e.g., in resource-poor settings with economical affordability and high mobility, different from most existing centrifugal microfluidic systems, a handheld centrifugal microfluidic platform with significantly shrunk size and cost has been developed. To achieve this goal, the traditional expensive, and bulky servo motor system with complicated feedback control was replaced by a low-cost, and small DC motor which is controlled by a flexible custom strategy. Meanwhile, the advanced real-time fluorescence detection system was replaced by simplified, endpoint visual detection by eyes. Specifically, to achieve the desired visual detection, a couple of different issues affecting visual discrimination between positive and negative tests were thoroughly analyzed. By taking advantage of the single rotation module equipped with a low-cost and small DC motor, sample distribution under high-speed centrifugation, accurate position switch between heating and detection positions, and multiplexed endpoint visual detection can be smoothly implemented based on an elaborate actuation mechanism. As a proof-of-concept, by combining a centrifugal chip with RPA, rapid and simple multiplexed molecular diagnosis of multiple ASFV genes can be conveniently achieved. 

In principle, besides the reagent, the performance of the handheld centrifugal microfluidic system can be further improved by optimizing the system design. For example, to improve the signal difference of visual detection between positive and negative tests by further reducing the background fluorescence from different respects. Accordingly, when the system background fluorescence is further reduced, satisfied visual detection can be easily achieved with even cheaper optical filters, which is beneficial to reducing system cost. Alternatively, instead of using visual observation, an upgraded optical module with photodiode-based fluorescence collection can be adopted to further extend the functionality and the application of the handheld device in POC testing. 

## Data Availability

Data are contained within the article.
